# Impact of cardiac rehabilitation on pre- and post-operative transcatheter aortic valve replacement prognoses

**DOI:** 10.3389/fcvm.2023.1164104

**Published:** 2023-12-13

**Authors:** Jieru Zou, Jie Yuan, Jingjin Liu, Qingshan Geng

**Affiliations:** ^1^ The Second Clinical Medical College, Jinan University, Shenzhen, Guangdong, China; ^2^ Department of Cardiology, Shenzhen People's Hospital (The Second Clinical Medical College, Jinan University; The First Affiliated Hospital, Southern University of Science and Technology), Shenzhen, Guangdong, China; ^3^ Department of Cardiology, Shenzhen Cardiovascular Minimally Invasive Medical Engineering Technology Research and Development Center, Shenzhen People's Hospital (The Second Clinical Medical College, Jinan University; The First Affiliated Hospital, Southern University of Science and Technology), Shenzhen, China; ^4^ Department of Geriatrics, Shenzhen People's Hospital (The Second Clinical Medical College, Jinan University, The First Affiliated Hospital, Southern University of Science and Technology), Shenzhen, Guangdong, China

**Keywords:** cardiac rehabilitation, transcatheter aortic valve replacement, aortic stenosis, frailty, exercise training

## Abstract

Transcatheter aortic valve replacement (TAVR) is a relatively new treatment method for aortic stenosis (AS) and has been demonstrated to be suitable for patients with varying risk levels. Indeed, among high-risk patients, TAVR outcomes are comparable to, or even better, than that of the traditional surgical aortic valve replacement (SAVR) method. TAVR outcomes, with respect to post-surgical functional capacity and quality of life, have also been found to be improved, especially when combined with cardiac rehabilitation (CR). CR is a multidisciplinary system, which integrates cardiology with other medical disciplines, such as sports, nutritional, mind-body, and behavioral medicine. It entails the development of appropriate medication, exercise, and diet prescriptions, along with providing psychological support, ensuring the cessation of smoking, and developing risk factor management strategies for cardiovascular disease patients. However, even with CR being able to improve TAVR outcomes and reduce post-surgical mortality rates, it still has largely been underutilized in clinical settings. This article reviews the usage of CR during both pre-and postoperative periods for valvular diseases, and the factors involved in influencing subsequent patient prognoses, thereby providing a direction for subsequent research and clinical applications.

## 1. Introduction

Valvular heart disease (VHD), most commonly caused by rheumatic (RHD) and degenerative conditions, is prevalent in developed countries. In fact, over the past 60 years in those countries, VHD has shifted from being predominantly due to RHD to degenerative diseases, though RHD remains the main cause in developing countries. Concerning RHD, its most frequent pathological outcomes are mitral valve insufficiency and aortic stenosis (AS) (1). For instance, in 2017, ∼12.6 million cases, and 102,700 deaths, from calcific aortic valve disease, of which a severe form is AS, were documented worldwide (2). More generally, VHD incidence and mortality have been gradually increasing as the global population ages.

With regards to AS, it has been associated with high mortality rates, if left untreated once symptoms appear. Two main treatment options have been developed, the traditional surgical aortic valve replacement (SAVR), and the newer transcatheter aortic valve replacement (TAVR); other, more conservative approaches, only tackle the symptoms and are inadequate substitutes for SAVR/TAVR. Since TAVR was first successfully executed among human patients in 2002 (3), it has become an established, popular procedure for AS patients, though concerns regarding post-surgical patient health management are still present. Cardiac rehabilitation (CR) has thus been considered a possible solution for such health management.

CR has been defined as a multidisciplinary collaborative program, which includes baseline patient assessment, exercise training, modification of cardiac risk factors, such as lipid levels, hypertension, weight, diabetes, and smoking, as well as psychosocial assessment and evaluation of outcomes (4). Furthermore, cardiac rehabilitation of the patient is a holistic strategy for optimal medical, physiological, psychological, social, and vocational performance following an acute cardiac event, and the upscaling of optimal medical therapy is part of the CR program. Timely medication adjustments as needed during cardiac rehabilitation (5). CR has been proven to be beneficial for cardiovascular disease patients, particularly for those with heart failure (HF) or coronary artery disease (CAD) (4, 5). However, the utility of CR for VHD still does not have definitive guideline recommendations. Furthermore, CR's impact on TAVR patient prognosis, especially for the pre-and post-operation periods, needs to be further defined. This review summarizes the current knowledge on the application of CR among TAVR patients, both pre-and post-operation, and the factors that influence subsequent prognoses, to provide possible directions for future studies and clinical applications.

## 2. CR use in TAVR

### 2.1. Current state of CR usage in TAVR

Since its first clinical application in France in 2002, TAVR has been established as the procedure of choice for elderly patients with severe AS and a high risk of perioperative death with SAVR (3), which yielded results comparable, or superior, to that of SAVR (6–11). TAVR also could serve as a viable treatment option for AS patients with clinical comorbidities (12), or multiple anatomical anomalies, particularly congenital ones, such as bicuspid aortic valves (13, 14). Furthermore, Makkar et al. showed that no significant difference was found in the incidence of death or disabling stroke 5 years after TAVR compared with surgical aortic valve replacement in patients with aortic stenosis who were at moderate surgical risk (15), indicating that it may become more accepted among younger patients with lower risk levels (7, 9, 16).

As more studies demonstrate the benefits of CR in alleviating valvular diseases (17, 18), more AS patients have been willing to undergo CR, particularly in conjunction with SAVR or TAVR. However, CR is still underutilized among both groups (19), with little change in overall participation over the years (20). And, currently, SAVR patients are more likely to undergo CR, not TAVR. Multiple reasons have contributed to this underutilization (20): (1) TAVR is a relatively new treatment modality; as a result, clear guidelines for recommending CR to TAVR patients have not been fully formulated, and few studies have been conducted to investigate the effectiveness of CR among these patients. Additionally, stereotypes regarding AS are still widely-held among physicians, such as excessive activity being recommended against, all of which contributes to the low referral rate for CR among AVR patients. (2) Compared to procedures requiring open surgery, TAVR patients have smaller incisions, shorter hospital stays, faster postoperative patient recovery, and a higher percentage of patients discharged to home, all of which contributes to a sentiment that CR is unnecessary for them. (3) Lack of clear influencing factors, such as age, preoperative health status, risk of depression, and comorbidities, where the lack of measurable effects of these variables can interfere with CR. (4) Hospital the TAVR patient is at may not have the relevant rehabilitation facilities. (5) Patient may not have health insurance coverage for CR. (6) Effect of the global coronavirus disease 2019 (COVID-19) pandemic (21). The COVID-19 pandemic prompted widespread dramatically reducing the delivery of non-essential outpatient services including CR (22, 23). All of these possibilities, stemming from uncertainties among physicians, patients, and their families, with respect to the effectiveness and feasibility of conducting CR in the context of TAVR have resulted in low referral rates for CR. In general, owing to increasing rates of VHD, and thus TAVR demand, it is predictable that CR utilization would likely increase, as part of optimizing post-surgical patient prognoses.

### 2.2. Pre-operative application of CR

Hospitalized patients with AS generally are severely symptomatic; as a result, they may be unable to perform physical activities or even be allowed to get out of bed. Owing to the physical status of the patient being a strong influence on post-TAVR prognosis, it would therefore be a point of interest to investigate whether pre-operative CR could affect this prognosis, which could, in turn, increase patient referrals for such rehabilitative procedures. Currently, insufficient evidence is present for pre-operative CR effectiveness, though it has been noted that the association of poor prognoses and decreased quality of life among cardiovascular disease patients, particularly with VHD, is more owed to the physical and mental functional status associated with cardiovascular defects, rather than the defects themselves (24).

As the population ages, more individuals will experience natural physiological frailty, which has been documented to be an independent predictor of cardiovascular disease prognosis. Concerning TAVR, frailty status was also a significant predictor for 1-year mortality post-surgery, compared to the separate Society of Thoracic Surgeons (STS) score, as shown by Rogers et al. (25). In the combined model with the European System for Cardiac Operative Risk Evaluation (EuroSCORE) and STS scores, Schoenenberger et al. found that the frailty index accounted for 58.2% and 77.6% of the predictive information, respectively. This suggests that the combination of the frailty index with conventional STS, as well as the EuroSCORE score, could significantly improve the predictive capability for 1-year mortality post-TAVR (26), compared to those scores alone. additionally, preoperative frailty assessment is expected to be valuable in distinguishing the development of new postoperative complications from simple exacerbation of pre-existing disease (27). Several recent studies have shown that preoperative frailty is associated with death, or poor short-term functional recovery post-SAVR/TAVR (28–31); in the long term, it is also associated with higher likelihoods of the aortic valve and HF-related hospitalizations (32). Therefore, this frailty could affect CR conduct, such as the initiation, type, intensity, frequency, and patient compliance with exercise training (33). Physicians at rehabilitation centers should thus collaborate with geriatricians to develop precise CR interventions for complex frail patients to ensure the most optimal survival prognoses, as well as determine whether using frailty measurements could improve outcomes for elderly and frail patients subjected to CR, particularly post-AVR. Aside from frailty, other geriatric syndromes elderly patients could present with, pre-operation, include cognitive deficits, severe dependency, and depression, all of which are strongly associated with longer postoperative hospital stays, as well as poorer functional and clinical outcomes after discharge (27, 34). Indeed, a study by Khan et al. found that the presence of cognitive deficits predicted postoperative delirium and mortality after TAVR (35), emphasizing the value of screening for geriatric risk factors before TAVR to identify high-risk patients.

Preoperative CR could also potentially improve functional capacity and shorten hospital stay lengths for patients undergoing surgery (36). TAVR patients generally have moderate to high-risk status and are thus vulnerable to perioperative respiratory infections. Weber et al. showed that pre-interventional inspiratory muscle training (IMT), though, significantly improved inspiratory muscle function, along with a 75% reduction in pneumonia, and a 25% reduction in hospitalization length among patients who underwent this physiotherapy during the perioperative period (37). IMT is an important preoperative intervention that could reduce the incidence of postoperative pulmonary complications (PPCs) (38, 39). Additionally, among younger, low-to-middle-risk TAVR patients, IMT has also been shown to facilitate postoperative recovery, in the form of improving functional capacity submaximal and inspiratory muscle strength (40, 41).

### 2.3. Post-operative application of CR

Regularly-scheduled exercises, along with other interventions, such as medications, are an established component of CR, and could significantly complement the effects of TAVR. Several studies have shown intensive post-operative recovery regimens, such as short-term exercise-based CR, especially for Phase I during hospitalization, could increase functional capacity, quality of life (42–44), and exercise tolerance, particularly with respect to the distance in the 6 min walk test (6MWT) (18, 45) and maximum workload (46–48). These effects are coupled with lowered hospitalization lengths (49), frailty (50), anxiety, and disability. As TAVR patients are older and more associated with non-cardiovascular comorbidities (51), postoperative CR may thus be beneficial in reducing non-cardiovascular-related mortality risks, such as infection or unintentional injuries (52). Indeed, two recent randomized controlled trials of IMT performed postoperatively have shown that conventional CR improved 6MWT results, which was augmented when combined with IMT (53, 54). IMT itself, as shown by Xu Lin et al., was able to improve exercise tolerance, pulmonary ventilation function, and inspiratory muscle strength, along with shortening postoperative hospital stay and reducing postoperative complications among TAVR patients. All of these outcomes appear to have sustained effects on improving survival times (54), though, the study was limited by its lack of evaluation on hard endpoints, such as patient readmission rates and mortality, as well as a short post-discharge follow-up of 3 months, and a high rate of patients lost to follow-up (35.4% at 3 months). Therefore, the long-term efficacy of IMT still needs to be explored in future studies by examining longer follow-up periods and using hard endpoints, such as readmission rates and mortality.

Exercise-based CR is an essential component for long-term comprehensive patient management post-TAVR. 6MWT is a relatively good evaluation method for CR, as it is simple, safe, effective, and able to be used at home to guide CR for TAVR patients (55–57). The gold standard, though, is the cardiopulmonary exercise test (CPET), which is more suitable for use in developing CR protocol if the patient has satisfactory post-operative indicators. With respect to the efficacy and safety of exercise-based CR, a randomized pilot trial has shown that an 8-week moderate combined endurance and strength training was safe, with no adverse effects on valve prosthesis, kidney, or neurohumoral function (58), and was able to improve long-term oxygen consumption at anaerobic threshold (VO_2_AT). However, peak VO_2_, muscle strength, or quality of life was unchanged (59), which contradicts findings from several other studies demonstrating that post-operative exercise was able to improve peak VO_2_ (60–62), though a longer follow-up period may be required to fully confirm the effects of exercise training on muscle strength and other quality of life indicators. Nevertheless, despite the risk of post-exercise complications, such as arrhythmias, musculoskeletal injuries, or chest pain, patients after TAVR can undergo CR safely and successfully if monitored and guided by rehabilitation physicians (61). Indeed, exercise did not affect aortic regurgitation severity or other valve functional parameters, suggesting that short-term postoperative endurance or resistance training was safe for valve integrity; these training procedures could even be conducted in elderly cohorts including patients in their 90s. Furthermore, A randomized controlled trial by Tamulevičiūtė-Prascienė et al. indicated besides general aerobic exercise, specially-tailored resistance/balance training is also well-tolerated among TAVR patients (48).

As for CR protocols, early implementation was found by Sire et al. to enhance patient exercise tolerance and quality of life, in which physical training shortly after AVR yielded rapid, sustained improvements in work capacity, without corresponding increases in cardiac load. However, the return to work was less influenced by training and socio-occupational assistance (63). Other case studies also showed that developing individualized, medically-supervised training programs safely and effectively returned the strength and fitness levels of young patients to that of preoperative levels (64). Along with the early implementation of post-operative training, the regimen used should be staged, going from simple to complex, passive to active, and bed to the floor, as well as gradually increasing exercise types, amounts, and duration.

Overview of the main clinical studies on cardiac rehabilitation after transcatheter aortic valve replacement is shown in Table 1.

**Table 1:** Overview of the main clinical studies on cardiac rehabilitation after transcatheter aortic valve replacement.

Year	Study	No. Patients	Type of Exercise	Frequency	Measures	Main findings
2023 (62)	Qiang Hu et al.	66	• moderate-intensity continuous training (MICT)	3 times/ week, for 3 months	• peak VO2 • 6MWT • SF-12	• Improved cardiopulmonary function • Improved physical capacity
2022 (54)	Lin Xu et al.	96	• CR program based on guideline recommendations	30 min/ d, 3–5 d/ week	• 6MWT, lung function • HGS, STS, QoL • functional status changes • arm-curl test	• improved exercise endurance • improved pulmonary ventilation function • improved inspiratory muscle strength • shorten the length of hospital stay
2021 (43)	Kleczynski et al.	105	• Treadmill (Bruce with ramp protocol) • Cycloergometer • functional exercise (Borg scale)	CR stay, 15–30 min/d, 6 d/week	• Qol(KI of ADL, HADS, KCCQ) • 5MWT, 6MWT • HGS	• Improved clinical performance • Improved QoL
2021 (45)	Penati et al.	46	• aerobic activities (Borg 10/20)	• 30 min (both), days 1 to 5;	• 6MWT • the SPPB scale • the Barthel scale	• Improved significantly all indices
			• group exercises alternating muscle strengthening, and stretching with balance exercises and coordination	• only one (6th day)		
				• for 6 d/week		
2021 (48)	Egle Tamulevičiūtė-Prascienė et al.	116	• endurance training (cycle ergometers)	6 times/ week	• exercise capacity (peak workload, peak VO2) • muscular strength (1RM) • functional capacity (6MWT) • physical performance (SPPB and 5MWT)	• Improved functional capacity • Improved physical performance • Improved exercise capacity • Improved muscular strength
			• aerobic dynamic gymnastics	30 min/d, 5 d/week		
			• respiratory muscle training	15 min/d, 7 d/week		
			• resistance and balance training	3 times/ week		
2019 (53)	Cargnin et al.	25	• Inspiratory muscle training	2 times/d, 7 d/week for 4 weeks	• Lung function • maximum inspiratory pressure (MIP) • 6MWT	• Improved inspiratory muscle strength • Improved lung function • Improved functional capacity
2019 (60)	Nilsson et al.	12	• aerobic exercise training	• 3 d/week • for 12 weeks	• peakVO2 • submaximal cardiopulmonary variables	• Improved significantly peakVO_2_
2019 (61)	Feier Song et al.	355	• Physical exercise (including walking, Tai chi, jogging, cycling, and brisk walking)	• 2 times/ week • 30 min/time	• 6MWT • VO2 peak • SF36	• Improved peakVO_2_
2018 (59)	Pressler et al.	27	• endurance and resistance training	8 weeks	• VO2AT, VO2peak • SF-12, KCCQ • 1RM	• Improved VO2AT

SF-12, the 12-Item Short Form Health Survey; 5MWT, 5 m walk time; KI of ADL, Katz index of Independence of Activities in Daily Living; 6MWT, 6 min walk test;.

HADS, Hospital Anxiety and Depression Scores; KCCQ, Kansas City Cardiomyopathy Questionnaire. SPPB, the short physical performance battery scale; HGS, hand.

grip strength; 1RM, One repetition maximum; VO2AT, anaerobic threshold; VO2peak, maximal oxygen uptake; SF36, standardized questionnaire Short Form-36.

## 3. Comparing CR impact between TAVR and SAVR

Compared to SAVR, less evidence exists on CR utilization among TAVR patients, in which recommendations have been outlined to implement CR as soon as possible among SAVR patients. However, the lowest-functioning AVR patients have been noted to be more likely to be female, older, possess higher STS, be classified with New York Heart Association (NYHA) classification III/IV, use more home oxygen, exhibit more comorbidities as well as having higher fall frequencies, mortality, and stroke disability rates, compared to those with more active functional statues (29, 32, 65). This patient demographic profile would more likely benefit from TAVR, but their greater frailty has made it more difficult to obtain CR evidence. Nonetheless, a meta-analysis found that CR yielded similar improvements in the Barthel index and 6MWD post-SAVR and TAVR (66). Therefore, the application of CR as soon as possible after surgery is also highly recommended for TAVR patients, as it would aid in obtaining higher quality of life and functional exercise capacity improvements.

## 4. Factors influencing AVR prognoses that require consideration when applying CR

### 4.1. Age

Aging, itself a risk factor, could affect post-surgical patient prognoses, in which TAVR patients tend to be older, as well as having more severe AS, comorbidities, and higher surgical risk; in particular, they are less able to tolerate open-heart surgery. A study from Attinger-Toller et al. found a linear trend between increasing age and all-cause mortality, stroke, and pacemaker implantation, during both the perioperative and long-term follow-up periods after TAVR (67). However, young patients are not risk-free from TAVR, as its risk is also related to the presence of comorbidities. Therefore, it is recommended, particularly for elderly patients, but also for young patients, to undergo CR post-TAVR, though the aging effect should also be taken into consideration for evaluating the effectiveness of CR.

### 4.2. Nutritional status

The nutritional status of AVR patients is also an important factor behind post-surgical recovery and CR effectiveness. For instance, absolute iron deficiency is a common occurrence post-cardiac surgery, being diagnosed in up to 10% of the patient population. It is associated with prolonged postoperative stays in the intensive care unit, along with lowered exercise tolerance and increased safety risks (68, 69). Additionally, many AS patients have diabetes, which is also an independent risk factor for post-surgical iron deficiency, illustrating the importance of plasma glucose levels on post-AVR recovery and the development of possible complications. As a result, routine screening for iron deficiency is strongly recommended for patients referred to the CR unit. In light of this phenomenon, rehabilitation physicians should account for the lowered functional capacity of patients with absolute iron deficiency post-AVR when developing individualized CR protocols, with particular attention paid to specific nutritional and pharmacological requirements.

Another example of the importance of considering nutritional status is demonstrated in a study conducted by Hebeler et al., which compared various functional and nutritional markers, such as gait speed, hand grip strength, serum albumin, and Katz Activities of Daily Living, in a pre-TAVR risk assessment analysis for 1-year mortality assessment. There, it was found that albumin was the sole marker associated with higher mortality (70), and that pre-operative adjustment of serum albumin levels could lower TAVR risk scores. Furthermore, this adjustment could exert a protective effect among obese patients (71–73), yielding pronounced improvements in 6MWT results post-surgery (46). However, this result contradicts observations from other studies (74); thus, further research may be required to fully validate this claim.

All of these findings thus indicate that aside from exercises and medications, nutritional therapy should be one of the 5 core components prescribed for CR, and that beyond routine general nutritional support, rehabilitation practitioners should also focus on individualized nutritional therapy. Furthermore, future studies should be carried out to determine whether optimizing specific nutritional statuses could improve outcomes after TAVR.

### 4.3. Gender

CR has long been underutilized among women, with men being ∼1.5 times more likely to be referred to rehabilitation centers (75), owing to differences in regional distributions, religious cultures, and economics. Furthermore, CR referral rates for AVR women are even lower. With respect to gender differences for AVR, the gender-specific risk of SAVR is independently associated with adverse prognoses (76, 77). On the other hand, TAVR women, compared to men, have lower 1-year mortality rates, despite having higher incidences of vascular and bleeding complications (78–81). Therefore, it may be more advisable to recommend TAVR for female patients with AS (82, 83). Indeed, A meta-analysis by Straiton showed that older women are more common among TAVR patients (16), and being a woman is an independent predictor for post-TAVR admission to rehabilitation facilities. This is due to women, pre-TAVR, being frailer than men (84), in turn serving as a major obstacle for post-TAVR functional recovery and subsequently increasing rehabilitation demands. This greater frailty also contributes to TAVR women having a higher in-hospital mortality rate vs. men (85), as highly-frail women pre-TAVR become even frailer pos-TAVR, leading to longer hospital stays, as well as increased complications, mortality, and other adverse outcomes (32, 86, 87). However, pre-operative rehabilitation approaches could lower the likelihood of such adverse outcomes among TAVR women. Therefore, it is strongly recommended to increase CR utilization among such female patients.

### 4.4. Psychological states

The psychological state of the patient also has a significant impact on post-TAVR recovery. Psychological disorders, such as depression and anxiety, are prevalent among AS patients prior to surgery (88, 89). Combining adverse psychological factors such as depression and anxiety increases the risk of poorer prognosis and death after cardiac events, increases the risk of short-term postoperative functional decline (90–92), prolongs postoperative hospital stays or increases readmission rates within 30 days of discharge, and decreases adherence to medications and recommended treatments (93). Pre-operative depression is an independent risk factor for death after heart valve surgery. Michael Ho et al. studied an unadjusted 6-month mortality rate of 13.2% in patients with depression (94). In addition, patients with moderate to severe anxiety before cardiac surgery present with higher postoperative pain scores and a significantly increased need for intra- and postoperative analgesia, according to Muhammad Kashif et al. (95). SAVR or TAVR, though, could aid in reducing anxiety among elderly patients with AS (89, 96) and increasing confidence in their bodily states (16, 97), while CR could aid in improving depressive states (18, 98–100). Screening for psychological disorders should be incorporated into preoperative risk stratification (101), and future research must determine whether interventions to treat psychological disorders preoperatively or postoperatively can improve outcomes. Studies have shown that one in five adults has moderate to very severe mental health symptoms at the time of entry into a CR program, and these patients are significantly less likely to complete a CR program (102, 103). The challenge is to identify those patients who may have difficulty recovering on their own, and refer them to rehabilitation centers so that they could promptly receive appropriate CR, to maximize their abilities to perform daily living activities and enhance their sense of well-being.

### 4.5. Effects of CR referrals and usage of alternative approaches to traditional CR

Delays in detecting symptoms, referrals (77, 104, 105), as well as denial of symptoms, are common among AS patients (106, 107), especially in women (84). These late referrals and refusal of surgery, and subsequently the lack of appropriate care, could result in poorer outcomes among patients with VHD, which is characterized by high morbidity and mortality. Heart valve clinics, therefore, could help patients identify these risk factors early, as well as determine the best times to undergo surgery, via risk stratification (108). With respect to CR, the COVID-19 pandemic has significantly decreased its global utilization (22), though, with recent advances, remote technologies have become increasingly capable of assisting with CR administration. These technologies could also be useful for high-risk TAVR patients, as they typically are severely frail and impaired in mobility and cognition, which renders traditional outpatient CR infeasible and necessitates the usage of other modalities to ensure engagement and compliance (65). In light of current studies or guidelines, remote technologies may also be used to facilitate community- or home-based CR (HBCR) (109–113).

Recruiting and referring older post-TAVR participants for CR has been demonstrated in the literature to be feasible (114) and safe (69). With respect to specific referral approaches, both center-based and HBCR had similar positive effects at the end of the intervention period, but HBCR was better at promoting long-term behavioral changes, subsequently yielding more lasting improvements after the active intervention ended (115). HBCR patients, post-TAVR, also had higher compliance and physical activity levels, especially among those who did not participate in traditional center-based CR. All these findings thus suggest that HBCR could address poor compliance with center-based rehabilitation, among a subset of patients, particularly in older adults.

### 4.6. Patient status post-discharge

Patients undergoing TAVR have a high rehospitalization rate, nearly 50% after 1-year. The most common causes of rehospitalization are HF and bleeding (116). Despite TAVR being able to significantly improve symptoms and quality of life among AS patients, postoperative patient mortality remains high, being 12.2% in France (117), 5.4% in Italy (118), and 12.4% in Germany (119), 30 days post-operation. Furthermore, in Germany, non-home discharge post-TAVR is associated with a high 1-year mortality risk (120). Multiple risk factors are associated with non-home discharge, including older age, non-transfemoral access, being female, frailty status, chronic pulmonary history, pacemaker placement, and insulin-dependent diabetes mellitus. These risk factors should be of particular concern when these patients are referred to the relevant rehabilitation center for treatment.

### 4.7. Smoking cessation

Cessation of smoking has been noted to be the most cost-effective strategy to prevent cardiovascular disease. As a result, smoking is prohibited in patients after AVR and should be mandated as part of CR strategies (121).

## 5. Conclusion

No definitive guidelines currently exist for recommended pre- and post-operative rehabilitative strategies for TAVR. However, based on the current findings, both pre-and post-operative CR, including exercise training, nutritional modifications, cession of smoking, and medications, under the supervision of trained medical professionals could be highly beneficial for TAVR patients, by further improving functional capacity and quality of life post-surgery. These studies, though, lack data comparing TAVR patients who have undergone CR vs. those who did not, making it difficult to distinguish whether the beneficial effects are due to symptomatic relief, or from CR participation. Furthermore, there is a lack of comparisons regarding the impact of receiving CR pre- vs. post-operatively. Therefore, future multicenter studies should be conducted to further differentiate the effects of CR in general, as well as pre-and post-operatively, on TAVR outcomes, along with promoting its utilization to positively influence factors affecting patient prognoses.
